# Total cholesterol and lipoprotein composition are associated with dry eye disease in Korean women

**DOI:** 10.1186/1476-511X-12-84

**Published:** 2013-06-05

**Authors:** Yoon Hong Chun, Hye Rang Kim, Kyungdo Han, Yong-Gyu Park, Ho Jin Song, Kyung-Sun Na

**Affiliations:** 1 Department of Pediatrics, Incheon St. Mary’s Hospital, College of Medicine, The Catholic University of Korea, Seoul, South Korea; 2 Department of Health Promotion Center, Seoul St. Mary’s Hospital, College of Medicine, The Catholic University of Korea, Seoul, South Korea; 3 Department of Biostatistics, The Catholic University of Korea, Seoul, South Korea; 4 Department of Ophthalmology and Visual Science, Seoul St. Mary’s Hospital, College of Medicine, The Catholic University of Korea, Seoul, South Korea

**Keywords:** Dyslipidemia, Hypercholesterolemia, Dry eye, Prevalence, Population-based study

## Abstract

**Background:**

This study aimed to determine the relationship between dyslipidemia and dry eye disease (DED) in a Korean population.

**Methods:**

This population-based study enrolled 5,627 adults (aged >19 years) who were participating in the first year of the fifth annual Korea National Health and Nutrition Examination Survey from 2010 to 2011. Clinically diagnosed DED and its symptoms were surveyed, and biochemical blood analysis data were collected. Dyslipidemia was defined as any of the following: hypercholesterolemia (total cholesterol > 200 mg/dL), hypertriglyceridemia (triglyceride > 150 mg/dL), low levels of high-density lipoprotein (<40 mg/dL), or high levels of low-density lipoprotein (>100 mg/dL).

**Results:**

After adjusting for demographics (age and body mass index), lifestyle (smoking, drinking, exercise, and residential district), and medical factors (diabetes, hypertension, previous ophthalmic surgery, menopause, and rheumatologic disease), elevated serum cholesterol level was found to be associated with increased likelihood of DED (odds ratio, 1.77; 95% confidence interval, 1.127–2.78) in women.

**Conclusions:**

DED in a Korean population was found to be associated with high serum cholesterol levelsThe results of this study highlight the significance of eye examinations and independent lipid profile monitoring in patients with dyslipidemia because of its possible correlation with DED progression.

## Background

The lipid layer is an essential component of the tear film, which maintains a smooth corneal surface and controls the evaporation rate from the eye [1]. The tear film lipid layer is composed chiefly of the meibomian glands, which are tubuloacinar holocrine glands that discharge their entire contents during the secretion process [2]. Chemical analysis of lipids secreted from normal meibomian glands shows that it consists of a mixture of non-polar lipids (wax esters, cholesterol, and cholesterol esters) and polar lipids (phospholipids and glycolipids) [3]. Systemic dyslipidemia, a disorder of lipid metabolism, may theoretically affect the meibomian lipid composition.

Dyslipidemia is one of the significant modifiable risk factors for cardiovascular diseases [4]. Total cholesterol (TC) is composed of 3 main types of lipoproteins—low-density lipoprotein (LDL), high-density lipoprotein (HDL), and very-low-density lipoprotein (VLDL) [4,5]. The association between elevated LDL cholesterol (LDL-C) and increased risk for cardiovascular events is well established [6], and other lipid parameters are also predictive of cardiovascular risk, including high non-HDL [4], low HDL [7], and elevated triglyceride (TG) levels [8].

Dry eye disease (DED) is a multifactorial disorder of the tears and the ocular surface that results in symptoms such as ocular discomfort, visual disturbance, and tear film instability with potential damage to the ocular surface [9]. Although the importance of the lipid layer in tear composition is well accepted, there is little information about whether dyslipidemia resulting from systemic lipid disorder is related to DED. Some epidemiologic data have reported conflicting results about such an association [10-13]. One recent animal study showed that lacrimal gland structure and function were differentially affected by changes in the lipid profile in mice [14]. A case–control study concluded that patients with moderate to severe meibomian gland disease (MGD), a major cause of evaporative DED, have a higher incidence of dyslipidemia with respect to elevated TC than the general population [12]. On the other hand, one prospective cohort study showed that although the presence of MGD does not correlate with dyslipidemia, the prevalence of high TG and LDL increases with increasing severity of MGD [13]. To make a more definite assessment of the association between dyslipidemia and DED, we assessed the data from the Korean National Health and Nutrition Examination Survey (KNHANES). Detailed data on lipid profile and DED were examined in the current study to determine the association between DED and dyslipidemia.

## Methods

### Study subjects and procedures

This cross-sectional study included a representative sample of the data gathered during the KNHANES V, which was performed from 2010 to 2011 by the Division of Chronic Disease Surveillance under the Korea Centers for Disease Control and Prevention.

The sampling units were registry households selected through a stratified multistage probability sampling based on geographic area, sex, and age group. Information was collected from stratified multistage probability samples of Korean households representing the non-institutionalized civilian population. The survey was composed of 3 parts, that is, a health interview survey, health examination survey, and nutrition survey, and it was a nationwide representative study of non-institutionalized civilians involving a stratified multistage probability sampling design using a rolling survey sampling model. Sampling units were defined based on household unit data from the 2010 National Census Registry, including those for geographic area, sex, and age.

Institutional review board/ethics committee approvals were obtained from the Catholic University of Korea in accordance with the Declaration of Helsinki. The data used here are publicly available from the Korean Centers for Disease Control and Prevention [15]. Written informed consent was obtained from the patient for publication of this report and any accompanying images. Questionnaires were used to collect demographic information, residential district (urban, rural), smoking history, educational status, alcohol consumption, medical history, and any use of prescription or nonprescription medications. Residential area was measured as urban versus rural, and urban included both large and small cities. Education level was measured according to 3 categories: less than high school, high school, and some college or higher. Household income was measured as quartiles based on inflation-adjusted per capita household income, which we used to classify individuals as being in the highest, middle-high, middle-low, and lowest quartiles. Based on the smoking behavior, individuals were categorized as current smokers, ex-smokers, or nonsmokers. Based on alcohol consumption, individuals were classified as nondrinkers, mild-to-moderate drinkers (1.0–30.0 g alcohol/day), or heavy drinkers (>30.0 g alcohol/day) after conversion of the average frequency and amount of alcoholic beverages consumed into the amount of pre-alcohol (in grams) consumed per day. Physical activity was divided into 3 groups according to frequency of exercise: ≤1 time per week, 2–3 times per week, and ≥4 times per week. KNHANES adopted the International Physical Activity Questionnaire to determine physical activity frequency. Menopause was defined as the participant’s self-reported menopause status or having had an hysterectomy.

Anthropometric measurements of the subjects were made by a specially trained examiner. Waist circumference was measured to the nearest 0.1 cm in a horizontal plane at the level of the midpoint between the iliac crest and the costal margin at the end of a normal expiration. Body mass index (BMI) was calculated as the individual’s weight in kilograms divided by the square of the individual’s height in meters. Systemic hypertension was defined as a measured systolic blood pressure of >160 mm Hg or a diastolic blood pressure of >90 mm Hg or the current use of systemic antihypertensive drugs.

Peripheral blood was obtained from each subject after fasting for at least 8 hours. Serum TC, HDL, and TG concentrations were enzymatically measured using a Hitachi Automatic Analyzer 7600 (Hitachi/Japan) with reagents (Pureauto SCHO-N, DAIICHI/Japan; CHOLESTEST N HDL, DAIICHI/Japan; Pureauto S TG-N, DAIICHI/Japan) by the NEODIN Medical Institute. The LDL-C concentration was calculated using the Friedewald equation (LDL − C = TC − [HDL-C + {TG ÷ 5}]) [16]. The prevalence of dyslipidemia was assessed according to the AHA and NECP-III report [17,18]. The cut-off value of dyslipidemia was any of the following: hypercholesterolemia (TC ≥ 200 mg/dL); hypertriglyceridemia (TG ≥ 150 mg/dL); low HDL (<40 mg/dL); or high LDL (≥100 mg/dL). Diabetes was defined as a measured fasting blood sugar of >126 mg/dL or current use of antidiabetic medication.

A DED questionnaire survey was conducted that included the following yes/no questions: (1) Have your eyes felt dry recently? and (2) Have you ever been diagnosed by an ophthalmologist as having DED? DED was defined as clinically diagnosed DED.

### Statistical analysis

The data are expressed as numbers and percentages (categorical) or mean ± standard error (continuous). Multivariable adjusted logistic regression analysis was conducted to examine the odds ratio (OR) and 95% confidence interval (CI) for the association of DED with TC, TG, HDL, and LDL. Age, education, residential area, smoking history, alcohol consumption, BMI, waist circumference, menopausal history (women), hypertension, and diabetes were used as covariates for calculating the adjusted OR.

Because the KNHANES V included weights to compensate for its complex sampling design and to allow approximations of the Korean population, weighted analyses were performed using SAS software (version 9.2; SAS Institute, Cary, NC, USA). Values of p < 0.05 were considered statistically significant.

## Results

Since Korea is a single-race nation, all of the subjects were Asian. Among 8,958 subjects in the first year of the KNHANES V, 8,141 (90.88%) underwent an ophthalmologic examination. We excluded the 1,858 who were aged <19 years, 449 whose DED data were missing, and 207 who had a blood test without an 8-hour fast. Of the remaining 5,627 individuals (2,408 men, 3,219 women), 116 men (5.06%) and 415 women (14.80%) were clinically diagnosed with DED.

The baseline characteristics of the study participants according to gender and DED are presented in Table 1. We found a significantly higher proportion of non-DED women residing in rural areas (p = 0.007). DED symptoms were more frequent in the DED group, both in men (p < 0.0001) and women (p < 0.0001). Both male (p < 0.0001) and female (p < 0.0001) subjects in the DED group had a statistically higher prevalence of any previous ophthalmic surgical history. The prevalence of DED was higher in men with a low BMI and small WC.

**Table 1:** Baseline characteristics of demographic, lifestyle, and medical factors of subjects according to gender and dry eye diseses

	**Male**			**Female**		
	**Non-DED (n = 2292)**	**DED (n = 116)**	**P-value**	**Non-DED (n = 2804)**	**DED (n = 415)**	**P-value**
Age (years)	44.2 ± 0.5	47 ± 1.9	0.2729	46.2 ± 0.5	44.9 ± 1.1	0.1566
Residential district (% rural)	21.8(3.3)	22.2(5.3)	0.9171	23.4(3.5)	14.4(3.1)	0.0007
Monthly income (% lowest quartile)	14.9(1.1)	13.3(3.8)	0.6809	19.3(1.2)	18.4(2.5)	0.7324
Education (% some college≥)	36.7(1.6)	37.9(5.7)	0.8314	28.9(1.4)	28.7(2.9)	0.9368
BMI (kg/m^2^)	24 ± 0.1	23.8 ± 0.3	0.0156	23.3 ± 0.1	22.7 ± 0.2	0.5919
Waist circumference (cm)	84.9 ± 0.9	84.2 ± 0.9	0.0063	78.1 ± 0.3	76.4 ± 0.6	0.593
Smoker (%)	42.3(1.2)	39.8(5.7)	0.648	5.5(0.6)	3.6(1.3)	0.2473
Heavy-drinker (%)	18.2(0.9)	13.3(3.7)	0.2571	1.8(0.3)	1.5(0.9)	0.7757
Exercise (% yes)	25.7(1.2)	23.8(5)	0.7217	20.3(1.1)	16(2.4)	0.1141
Menopause (% yes)	NA	NA	NA	35.5(1.5)	38.3(3.1)	0.4255
Symptoms of DED (% yes)	8(1)	81.3(4.7)	<.0001	12.1(1.1)	81.4(3)	<.0001
Diabetes (% yes)	11(0.9)	10.9(3.3)	0.9851	9.1(0.8)	8.9(1.6)	0.8944
Hypertension (% yes)	31(1.5)	27.3(5.7)	0.5244	46.5(1.4)	48.3(3.3)	0.6178
Rheumatoid arthritis history (% yes)	1.6(0.4)	1.4(1.1)	0.8616	3.1(0.4)	3.2(0.9)	0.9261
Any ophthalmic surgery (% yes)	7.8(0.6)	24.6(5.1)	<.0001	13.5(0.8)	24.1(2.4)	<.0001
Mean SBP (mmHg)	122 ± 0.5	121.2 ± 1.7	0.3422	117.4 ± 0.6	116.3 ± 1	0.6275
Mean DBP (mmHg)	80.4 ± 0.4	78.7 ± 0.9	0.8309	74.3 ± 0.3	74.2 ± 0.5	0.0868
Laboratory data						
Glucose (mg/dL)	98.2 ± 0.7	98.1 ± 1.9	0.8866	94.3 ± 0.5	94.1 ± 1.2	0.9478
Insulin (uIU/mL)	10.4 ± 0.1	9.9 ± 0.4	0.375	10.5 ± 0.1	10.1 ± 0.2	0.3519
Total cholesterol (mg/dL)	187.2 ± 1	184.5 ± 3.4	0.6799	186.7 ± 0.9	185.8 ± 2.2	0.4302
Triglycerides (mf/dL)	157.4 ± 3.9	134 ± 9.7	0.721	109.3 ± 2	106.2 ± 4	0.1337
HDL	49.6 ± 0.3	51.5 ± 1.5	0.6395	56 ± 0.3	56.3 ± 0.7	0.2197
LDL	112.3 ± 0.9	110.4 ± 3.3	0.8224	113.9 ± 0.7	113.4 ± 1.9	0.5698

Values are represented as means ± SD. Numbers in brackets represent percentages (standard error).

DED = dry eye disease; BMI = Body mass index; SBP = systolic blood pressure; DBP = diastolic blood pressure; HDL = high-density lipoprotein; LDL = low-density lipoprotein.

**Figure 1:**
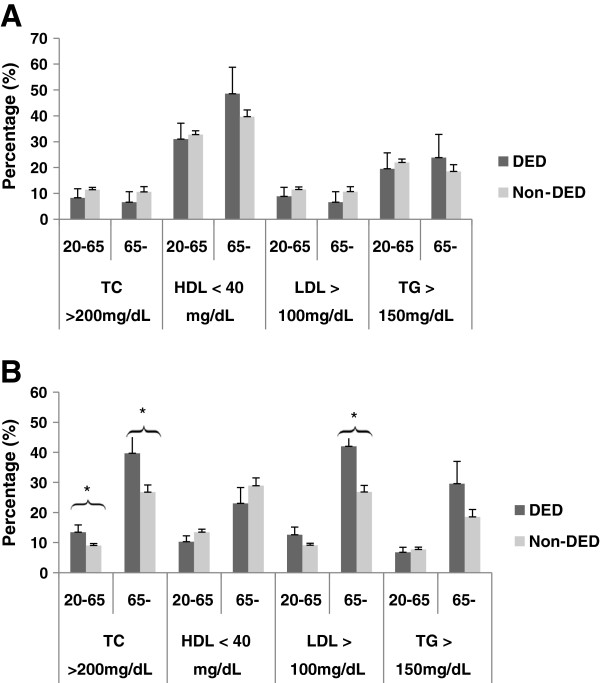
**Prevalence of dyslipidemia with respect to TC, HDL, LDL, and TG in patients with DED and normal control subjects stratified by age in male (A) and female (B).** There were statistically significant differences the prevalence of high cholesterol and low-density lipoprotein levels between female DED patients aged >65 years and those without DED. The differences in high cholesterol prevalence seen in patients aged 20–65 years were also statistically significant. TC, total cholesterol; HDL, high-density lipoprotein; LDL, low-density lipoprotein; TG, triglycerides; DED, dry eye disease. *Statistically significant difference between the 2 groups. The cut-off value for dyslipidemia was any of the following: hypercholesterolemia (TC ≥ 200 mg/dL), hypertriglyceridemia (TG ≥150 mg/dL), low levels of HDL (<40 mg/dL), or high levels of LDL (≥100 mg/dL).

In patients with DED stratified according to the age group (20–65 and >65 years), there were statistically significant differences in the prevalence of high cholesterol and LDL between female patients with DED aged >65 years and normal female control subjects. Statistically significant differences in high cholesterol prevalence were also seen among those aged 20–65 years (Figure 1).

**Figure 2:**
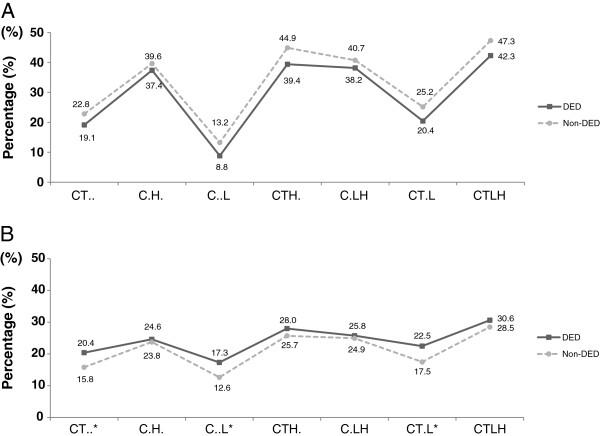
**Comparison of the prevalence (%) of different combinations of dyslipidemia in patients with dry eye disease (DED) and those without DED. A**, male subject; **B**, female subject; DED ■; non-DED (gray circle), C, hypercholesterolemia (total cholesterol ≥ 200 mg/dL); T, hypertriglyceridemia (triglyceride ≥150 mg/dL); H, low high-density cholesterol (HDL) < 40 mg/dL; L, high low-density cholesterol (LDL) ≥ 100 mg/dL. * Statistically significant difference between the 2 groups. The definition of dyslipidemia was the prevalence of any of the following conditions: hypercholesterolemia (TC ≥ 200 mg/dL); hypertriglyceridemia (TG ≥ 150 mg/dL); low HDL (<40 mg/dL); or high LDL (≥100 mg/dL).

We detected some differences in prevalence after stratification according to different combinations of independent lipid profiles. The prevalence of the 3 combinations, hypercholesterolemia + hypertriglyceridemia, hypercholesterolemia + high LDL-C, and hypercholesterolemia + hypertriglyceridemia + high LDL-C, were significantly different between women with DED and women without DED (Figure 2).

Univariate analysis revealed that the lipid profile factors associated with clinically diagnosed DED were high cholesterol. There was a statistically smaller percentage of low HDL in female diagnosed DED and who has symptoms of DED, when compare to control (Table 2).

**Table 2:** **Prevalence of dyslipidemia**^Φ ^**in patients of dry eye diseases and normal control by lipid profiles in Korean population**

**Clinically diagnosed DED**						
	**Male**			**Female**		
	**No**	**Yes**	**P-value**	**No**	**Yes**	**P-value**
Hypercholesterolemia	11.3(0.8)	8(3)	0.351	11.7(0.7)	16.2(2.3)	0.033
Hypertriglycemia	21.5(1.2)	20.3(5.6)	0.8313	9.5(0.7)	9.6(1.7)	0.9595
Low HDL	33.5(1.4)	33.6(5.4)	0.9909	15.8(0.9)	11.7(1.7)	0.0461
High LDL	11.4(0.9)	8.5(3)	0.413	11.8(0.7)	15.7(2.5)	0.0921
**Symptoms of DED**						
					Male			Female	
	No	Yes	P-value	No	Yes	P-value			
Hypercholesterolemia	10.8(0.8)	12.6(2.5)	0.4816	11.8(0.8)	14.8(1.7)	0.095			
Hypertriglycemia	21.4(1.3)	25(3.8)	0.3326	9.6(0.8)	8.9(1.4)	0.6573			
Low HDL	33.3(1.4)	29.7(3)	0.2591	16.3(1.1)	11.6(1.5)	0.0215			
High LDL	11.1(1)	12(2.4)	0.7271	12(0.7)	14.8(1.8)	0.1232			

Data are expressed as percentage (standard error).

Φ The cut-off value of dyslipidemia was as any of the following : hypercholesterolemia (TC ≥ 200 mg/dL); hypertriglycemia (TG ≥150 mg/dL); low HDL, <40 mg/dL; or high LDL ≥ 100 mg/dL.

After adjustment for demographics (age, BMI), lifestyle (smoking, drinking, exercise, and residential district), and medical factors (diabetes, hypertension, previous ophthalmic surgery, menopause, and rheumatologic disease), the prevalence of DED was calculated by gender, and each calculated OR had a 95% CI with respect to lipid profiles. The lipid profile associated with increased likelihood of DED was elevated cholesterol level (OR, 1.77; 95% CI, 1.127–2.78) in women (Table 3).

**Table 3:** Odds ratio (95% CI) on multivariate analysis for anlalyzing association between lipid profiles and dry eye diseases in Korean population

	**Hypercholesterolemia**	**Hypertriglycemia**	**Low HDL**	**High LDL**
**Male**				
MODEL1^†^	0.677(0.308,1.488)	0.94(0.477,1.853)	1.006(0.635,1.593)	0.704(0.328,1.511)
MODEL2^‡^	0.676(0.307,1.491)	0.943(0.467,1.905)	1.024(0.652,1.608)	0.7(0.325,1.505)
MODEL3^§^	0.588(0.233,1.48)	1.008(0.482,2.106)	1.258(0.771,2.05)	0.614(0.259,1.457)
**Female**				
MODEL1^†^	1.846(1.244,2.739)	1.126(0.717,1.766)	0.793(0.543,1.159)	1.731(1.102,2.719)
MODEL2^‡^	1.795(1.21,2.661)	1.173(0.742,1.854)	0.812(0.555,1.187)	1.687(1.07,2.66)
MODEL3^§^	1.77(1.127,2.78)	1.241(0.719,2.141)	0.697(0.414,1.174)	1.521(0.893,2.59)

The cut-off value of dyslipidemia was as any of the following : hypercholesterolemia (TC ≥ 200 mg/dL); hypertriglycemia (TG ≥150 mg/dL); low HDL, <40 mg/dL; or high LDL ≥ 100 mg/dL.

† Model1 = Adjusted for demographic factors (age, and body mass index)

‡ Model2 = Model1 + adjusted for lifestyle factors (smoking, drinking, exercise, and residential district)

§ Model3 = Model 2 + adjusted for medical factors (diabetes, hypertension, previous ocular surgery, menopause, and rheumatologic diseases).

## Discussion

To our knowledge, the current study is the first population-based study in Korean to reveal that female subjects with DED had increased rates of hypercholesterolemia compared to normal female controls. Earlier reports of dyslipidemia and DED showed conflicting results.

One retrospective control study of 66 patients concluded that patients with moderate to severe MGD had a higher incidence of dyslipidemia with respect to elevated cholesterol than the general population as reported by data from the National Health and Nutrition Examination Survey (NHANES) [12]. When stratified according to gender, only men aged 20–44 and 45–64 years showed a statistically different levels of hypercholesterolemia. They found the component of total cholesterol that contributed most to increase in total cholesterol came from elevated serum HDL levels. This study has some limitations due to its relatively small sample size and an overall population that was older than the normal population in the NHANES. Moreover, menopausal history, which greatly affects the development of DED, was lacking in the NHANES.

Another Taiwan population-based study also found that the prevalence of hyperlipidemia was significantly higher in patients with DED [11]. After adjusting gender, age, and socioeconomic status, they showed that compared to normal control, patients with DED had a greater tendency to have hyperlipidemia.

Unlike these results, in the Beaver Dam Eye study, Moss et al. found that the incidence of DED was not significantly associated with serum total cholesterol, HDL, or cardiovascular diseases after controlling age and gender in population-based cohort [10].

The current study revealed that only female patients with DED had a statistically higher prevalence of hypercholesterolemia. The reason hypercholesterolemia occurs in the presence of DED can be explained as increased cholesterol in the meibomian lipid would increase its melting point to 46°C versus the normal meibomian lipid melting point of 30–34°C and, thus, lead to increased viscosity and plugging of the meibomian orifice [19].

Interestingly, we found that low HDL was more prevalent in female subjects with controls than that in female with DED, which was statistically significant. It is quite surprising that HDL, which is well known for its preventive effect in cardiovascular disease, may acts negatively in the presence of DED. Among female subjects, none of the combinations with low HDL-C demonstrated significant differences in prevalence between the DED and non-DED groups, whereas every combination without high HDL-C showed significant differences. This finding may explain the opposing effects of HDL in DED development and cardiovascular diseases. The mechanism why elevated HDL may be a risk factor for the development of MGD despite its cardioprotective role is not clear. Meibomian glands, a modified form of sebaceous glands, are reported to be uniquely related to cholesterol biosynthesis [19]. A microsomal enzyme, acyl-CoA:cholesterol acyltransferase (ACAT) catalyzes the esterification of cellular cholesterol, which is subsequently secreted into the circulation in the form of lipoproteins [20]. A murine model study showed that HDL and VLDL markedly enhanced sebaceous epithelial cell differentiation, which is defined by the increasing accumulation of lipid droplets, the major component of sebum [20]. The absence of ACAT-1 was shown to attenuate atherosclerosis but cause meibomian gland atrophy and cutaneous xanthomatosis in a mouse model [21].

One randomized trial of healthy male and female subjects reported that simvastatin, which reduces blood cholesterol levels, was associated with meibomian gland obstruction and skin sebaceous gland hypotrophy [22]. HDL transports cholesterol from the tissues to the liver to be disposed, making it beneficial for cardiovascular disease; however, it is plausible that HDL stimulates lipid production within the meibomian gland to alter its melting point and decrease its viscosity.

In the current study, MGD was not assessed in patients with DED, so it may include a combination of DED types. However, the International Workshop on Meibomian Gland Dysfunction has reported a striking feature that the prevalence of MGD appeared to be higher in studies on Asian populations [23]: 46.2% in a Bangkok study [24], 60.8% in the Shipai Eye Study [25], 61.9% in a Japanese study [26], and 69.3% in the Beijing Eye study [27]. Although the nationwide prevalence of MGD has not yet been reported in Korea, we assume that more than half of the patients who report having clinically diagnosed DED also have MGD, considering the previous epidemiologic studies in Asia.

Our study has some potential limitations. The KNHANES data were not collected for ophthalmologic evaluation; hence, much of the information was self-reported and recall bias may exist. Additionally, no objective diagnostic testing of dry eye was performed. However, with regard to public health, subjective methods such as these are valuable for DED studies because symptoms directly affect the quality of life [9]. The strengths of this study are that it is the first large population-based study in Korea and that it elucidated the relationship between dyslipidemia and DED. In addition, the anthropometric measurements and diabetes and hypertension diagnoses were made by trained examiners and physicians in contrast to earlier studies, which based this information on self-reporting. Finally, since Koreans have relatively uniform genetic and environmental influences including a single race, climate, and food culture, our results may be more consistent than those of other large population-based studies. There have been few studies in high-income Asian countries, and it would be interesting to compare their results to those of low- and middle-income Asian countries, where the prevalence of DED and dyslipidemia differ.

In conclusion, after adjustment for systemic and socioeconomic parameters, DED in this Korean population was associated with high serum cholesterol level but not with LDL, or TG levels in female subjects. And HDL may be a risk factor for DED despite of its cardiovascular protective effect. However, further verification of both prospective and basic science studies, would be needed. The results of this study highlight the significance of eye examinations and lipid profile monitoring in dyslipidemia patients due to its possible correlation with the progression of DED. The systemic lipid transporter system may affect local lipid biosynthesis, although differently than that in cardiovascular diseases. Further studies are needed to confirm the exact mechanism and correlation between DED and dyslipidemia.

## Competing interests

The authors declare that they have no competing interests.

## Authors’ contributions

YHC analyzed the data and wrote the manuscript. HRK and HJS participate in the execution and analysis of this study. KDH and YKP participate the statistical analysis. NKS designed and critically revised the manuscript. All authors read and approved the final manuscript.
